# A new species of genus *Monoctonus* (Hymenoptera, Braconidae, Aphidiinae) from South Korea

**DOI:** 10.3897/BDJ.12.e119476

**Published:** 2024-04-15

**Authors:** Sangjin Kim, Jelisaveta Čkrkić, Željko Tomanović, Ju-Hyeong Sohn, Jongok Lim, Hyojoong Kim

**Affiliations:** 1 Kunsan National University, Gunsan, Republic of Korea Kunsan National University Gunsan Republic of Korea; 2 University of Belgrade, Belgrade, Serbia University of Belgrade Belgrade Serbia; 3 Centre for Biodiversity Genomics, University of Guelph, Guelph, Canada Centre for Biodiversity Genomics, University of Guelph Guelph Canada; 4 Serbian Academy of Sciences and Arts, Belgrade, Serbia Serbian Academy of Sciences and Arts Belgrade Serbia; 5 Wonkwang University, Iksan, Republic of Korea Wonkwang University Iksan Republic of Korea

**Keywords:** DNA barcoding, parasitoid wasps, systematics, taxonomy

## Abstract

**Background:**

The genus *Monoctonus* Haliday, 1833 is a small group which consists of 24 species worldwide. In South Korea, Chang and Youn (1983) recorded one species, *M.similis* Starý & Schlinger, 1967, but the evidence for identification of this species is doubtful and further confirmation is required (personal communication with Prof. Jong-Cheol Paik).

**New information:**

An additional *Monoctonus* species is recorded as new to science from South Korea. Descriptions and illustrations of the new species –*Monoctonuskoreanus* sp. nov. – are provided, together with its mitochondrial *cytochrome c oxidase subunit I* (*COI*) data and phylogenetic position. A key to the female of the two species present in Korea is provided.

## Introduction

The genus *Monoctonus* Haliday, 1833 consists of 24 species (Čkrkić et al. 2020, Tomanovic et al. 2023) worldwide and belongs to the subtribe Monoctonina Mackauer, 1961, which also includes genera *Falciconus* Mackauer, 1959, *Harkeria* Cameron, 1900, *Monoctonia* Starý, 1962 and *Quadrictonus* Starý and Remaudière, 1982 (Yu et al. 2016, Čkrkić et al. 2019, Čkrkić et al. 2020, Tomanovic et al. 2023). In South Korea, Chang and Youn (1983) recorded one species, *M.similis* Starý & Schlinger, 1967, but the evidence for identification of this species is doubtful and further confirmation is required (personal communication with Prof. Jong-Cheol Paik).

*Monoctonus* species are solitary koinobiont endoparasitoids of aphids, but are rarely used as biological control agents, because their host plants are mostly not economically important (Čkrkić et al. 2019). Most species in the genus *Monoctonus* are known as parasitoids of aphids in Aphidini and Macrosiphini (Yu et al. 2016), except for *M.gallicus* Starý, 1977 which parasitises a Panaphidini species, *Therioaphisriehmi* (Börner) (Starý et al. 1977). Members of the genus have a ploughshare-shaped ovipositor sheath, ranging from moderately widened ventrally to distinctly ploughshare-shaped (Čkrkić et al. 2019).

Within the subtribe Monoctonina, the genus *Monoctonia* is separated from other species used in the analysis of the barcode region by Rakhshani et al. (2015): *Monoctonusligustri* van Achterberg, 1989, *M.crepidis* (Haliday, 1834), *Harkeriaangustivalva*, (Starý, 1959) and *Falciconuspseudoplatani*, (Marshall, 1896). A subsequent study employed two molecular markers (COI and 28S rDNA) and morphological data and analysed most species of Monoctonina (Čkrkić et al. 2020). The genera *Falciconus* and *Monoctonia* form a separate basal clade, while *Harkeriaangustivalva* is combined with the genus *Monoctonus* (Čkrkić et al. 2020).

Amongst the *Monoctonus* species, most species are distributed in the Holarctic, with the exception of the oriental *M.fotedari* Bhagat, 1981 and *M.woodwardiae* Starý & Schlinger, 1967. Two of the remaining 22 species are distributed throughout the Holarctic and 10 species are distributed in the Nearctic and Palaearctic each. Only two species are distributed in the East Palaearctic, with only one host recorded for each of them (Yu et al. 2016, Čkrkić et al. 2019, Čkrkić et al. 2020, Tomanovic et al. 2023). Furthermore, because the record in Soth Korea is doubtful (personal communication with Prof. Jong-Cheol Paik), information on this genus in Korea is very scarce.

In this study, we describe and diagnose a new species, *M.koreanus* sp. nov. and analyse its phylogenetic relationships within the genus using the *COI* barcoding region.

## Materials and methods

### Sample collection and morphological identification

The sample was borrowed from the Korean National Arboretum. It was obtained with a Malaise trap and collected in South Korea. It was stored in 95% ethyl alcohol at -19℃. The identity and morphological characters of the specimen were compared with those described in Starý and Schlinger (1967), Starý and González (1978), Bhagat (1981), van Achterberg (1989), Jorge Tizado (1992), Pike et al. (2000), Tomanović et al. (2002), Čkrkić et al. (2019) and Čkrkić et al. 2020. We used a dissecting microscope (OLYMPUS SZX16, Leica M205C, NIKON SMZ 1500), followed by a DNA extraction.

### DNA experiment

DNA extraction was performed using a LaboPass Tissue Genomic DNA Isolation Kit mini (COSMO genetech, Daejeon, Korea) following the manufacturer’s protocol. To preserve a morphologically complete specimen, the DNA extraction method was slightly modified from the “freezing method” by Yaakop et al. (2013). In the original protocol, the sample was homogenised, and then incubated 30 minutes at 56°C with 200 μl of TL buffer + 20 μl of proteinase K. In slightly modified DNA extraction methods, before the incubation with buffers, samples were heated for one hour at 70°C with 1,000 μl of distilled water (DW). After DW was removed, 200 μl of TL buffer + 20 μl of proteinase K were added without destroying the sample, followed by 20 minutes of incubation at 55°C. Samples in this condition were then kept in a freezer at -22°C overnight. After that, the general protocol was used for the remaining steps. The target site for molecular identification was the front partial region of mitochondrial *COI*, viz. a 658-bp fragment, amplified using primers LCO1490 (forward) 5’-GGTCAACAAATCATAAAGATATTGG-3’ and HCO2198 (reverse) 5'-TAAACTTCAGGGTGACCAAAAAATCA-3’ (Folmer et al. 1994) and AccuPower PCR PreMix (Bioneer Corp., Daejeon, Korea). Polymerase chain reaction (PCR) amplification was conducted with 20 μl of a reaction mixture consisting of 3 μl of DNA extract, 2 μl of primer and 15 μl of DW. It was carried out as follows: denaturation for 5 min at 95℃; 35 cycles of 60 s at 94℃, 60 s at 54℃, 90 s at 72℃; and final extension at 72℃ for 7 min. PCR products were tested by electrophoresis on agar gel and, if a band existed, we commissioned BIONICS (Seoul, Korea) for Sanger sequencing and purification.

### Data analysis

The 658 bp barcode region of the *COI* gene was sequenced from the examined specimen and deposited in GenBank. BOLD identification System (Ratnasingham and Hebert 2007) was used to check the generated sequence against the BOLD reference library for possible matches. Altogether, 47 sequences of 18 Monoctonina species, containing three species, for which only molecular data and morphology of males are known as *Monoctonus* sp. 1, 2, 3 in Čkrkić et al. (2020), were retrieved from GenBank and BOLD (http://www.boldsystems.org) and were used to compare them with *M.koreanus* sp. nov.. *Aphidiustranscaspicus* was used as an outgroup (Suppl. material 1).

Using MEGA, version 7.0 (Kumar et al. 2016), sequences were aligned by ClustalW default settings and their frame-shifts checked to avoid pseudogenes. Alignment was translated to amino acids to confirm the codon position. We calculated sequence divergences using the ‘*p*-distance’ model commonly employed to analyse *COI* barcoding data. A phylogeny tree was constructed using the Neighbor-joining method with 1,000 bootstrapping replications and pairwise deletion in data gaps.

### Taxonomic work

Measurements of the new species were carried out. A Dhyana 400DC digital camera (Tucsen, Photonics Co., Ltd., Fuzhou, China) and a LEICA M205 C microscope (Leica Geosystems AG, Wetzlar, Germany) were used for photography and characterisation, several pictures being taken for each height using multifocusing technology. Helicon Focus 7 (Helicon Soft, Kharkiv, Ukraine, https://www.heliconsoft.com) software was used for stacking work. Mosaic V2.2 (Tucsen, Photonics Co., Ltd., Fuzhou, China) was used to ascertain the shape of specimens (Berkovitch et al. 2009).

## Taxon treatments

### Monoctonus koreanus

Kim, Čkrkić & Tomanović sp. nov.

69EBEC19-0278-5FB8-BEAF-E7E1C0B693AB

OK641850

345CBFD6-4245-4CAB-B54A-95478D17840E

#### Materials

- **Type status:** Holotype. **Occurrence:** individualCount: 1; sex: female; lifeStage: adult; reproductiveCondition: 95% ehanol; occurrenceStatus: present; occurrenceID: 46F8464F-6708-5C83-B14C-733C9814325B; **Taxon:** scientificName: Monoctonuskoreanus Kim, Čkrkić & Tomanović; kingdom: Animalia; phylum: Arthoropoda; class: Insecta; order: Hymenoptera; family: Braconidae; genus: Monoctonus; specificEpithet: koreanus; scientificNameAuthorship: Kim, Čkrkić & Tomanović, 2024; **Location:** higherGeography: East Asia; country: South Korea; countryCode: KR; stateProvince: Gangwon-do; municipality: Yanggu-gun; locality: Songhyeon-ri; **Identification:** identifiedBy: Sangjin Kim, Jelisaveta Čkrkić, Željko Tomanović, Ju-Hyeong Sohn, Jongok Lim, Hyojoong Kim; **Event:** eventDate: 24/09-27/10/2020; year: 2020

#### Description

**Figure 1.**
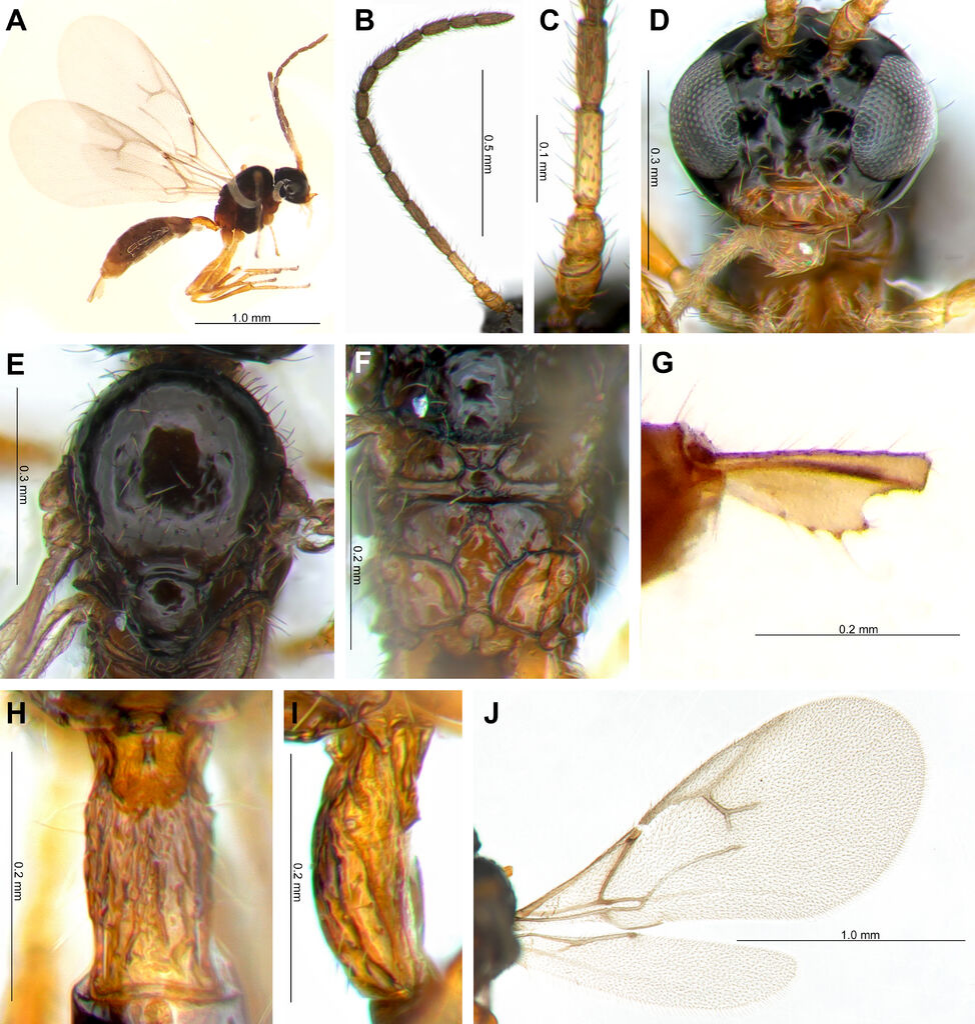
*Monoctonuskoreanus* sp. nov. Kim, Čkrkić & Tomanović, Holotype female: **A** Body; **B** Antenna; **C** F1 and F2; **D** Head; **E** Mesoscutum; **F** Propodeum; **G** Ovipositor; **H** Dorsal view of petiole; **I** Lateral view of petiole; **J** Wings.

**Female.** Length of body about 2.2 mm (Fig. 1A). Length of fore-wing 1.9 mm (Fig. 1J).

**Head.** Tentorial index 0.35 (Fig. 1D). Clypeus oval with eight long setae. Malar space 0.2 × longitudinal eye diameter. Antenna 15-segmented (Fig. 1B). F1 longer than F2 (F1/F2 length 1.2). F1 and F2 3.9 and 2.5 times as long as their width at the middle, respectively. Both F1 and F2 with three longitudinal placodes (Fig. 1C). Maxillary palp with four palpomeres, labial palp with three palpomeres. Temple subequal to eye in dorsal view. Face width/height ratio 1.1 (Fig. 1D).

**Mesosoma.** Mesoscutum with effaced notaulices, dorsal surface smooth with numerous setae (Fig. 1E). Head width/mesoscutum width ratio 1.2. Propodeum areolated, central areola length/width ratio 1.5 (Fig. 1F). Fore-wing pterostigma 5.7 times as long as wide. Ratio of pterostigma length to R1 vein (= metacarpus) length 3.2 (Fig. 1B). Vein m-cu distinct, 2RS visible in half, veins r and 3RS distinct.

**Metasoma.** Petiole 2.0 times as long as wide at spiracles (Fig. 1H, I). Dorsal surface rugose with prominent dorsal carinae (Fig. 1H). Ovipositor sheath distinctly ploughshare-shaped. Ovipositor sheath length/width ratio 2.4.

**Colour.** F1 and basal ¼ to ½ of F2 yellow, remainder of antenna brown. Scape and pedicel yellowish-brown. Head and face black. Clypeus yellowish-brown. Mouthparts dark yellowish-brown. Dorsal side of mesoscutum and metasoma dark brown, except light brown propodeum and yellowish-brown petiole. Legs yellowish-brown.

#### Diagnosis

In some morphological characters (shape of the ovipositor sheath, shape of the first flagellomere and antenna, number of antennal segments and number of maxillary and labial palpomeres), *M.koreanus* sp. nov. is similar to *M.brachyradius*, *M.nervosus* and *M.paulensis*. However, it clearly differs from them in having a broad ovipositor sheath (ovipositor length/width is 2.4 in *M.koreanus* sp. nov., while 3.0 in *M.brachyradius*, 2.6–2.8 in *M.nervosus* and 2.7 in *M.paulensis*). Additionally, having a longer distal abscissa of R1 vein than in *M.brachyradius* (pterostigma length/R1 length is 3.2 in *M.koreanus* sp. nov., while 4.8–5.1 in *M.brachyradius*) and shorter than in *M.nervosus* and *M.paulensis* (1.8–2.3 in *M.nervosus*, 2.1–3.0 in *M.paulensis*) and a higher number of longitudinal placodes in flagellomere 1 (three in *M.koreanus* sp. nov., while no placodes in other species). It differs from *M.brachyradius* and *M.nervosus* in possessing a less elongate pterostigma (pterostigma length/width ratio is 5.7 in *M.koreanus* sp. nov., while 7.5 in *M.brachyradius* and 6.1–6.7 in *M.nervosus*) and a higher tentorial index (tentoriocular line length/intertentorial line length ratio is 0.35 in *M.koreanus* sp. nov., while 0.1 in *M.brachyradius* and 0.2 in *M.nervosus*). Although the petiole is an important character, most known *Monoctonus* species having a short petiole length/width ratio at spiracles about 2.0 contain *M.koreanus* sp. n.), except for *M.canadensis* Čkrkić, Petrović & Tomanović, 2019, *M.inexpectatus* Čkrkić, Petrović & Tomanović, 2019 and *M.montengrinus* Petrović & Tomanović, 2023, in which these ratios are about 2.5, 2.3 and 2.6, respectively.

#### Etymology

The new species is named, based on its current known distribution (Republic of Korea).

#### Notes

Although the new species is morphologically most similar to members of the *nervosus* group s.s., molecular data place it closer to other members of the *nervosus* group s.l. (Čkrkić et al. 2019). Since only one specimen was used in this description, it would be advisable to collect more samples and investigate potential intraspecific morphological and molecular variability, as well as aphid hosts of the new species.

## Identification Keys

### Key to female *Monoctonus* in South Korea

**Table:** 

1	Antenna with 13 antennomeres; pterostigma length/width ratio about 3.0, pterostigma length/R1 vein ratio about 3.0, 2RS and m-cu vein absent	*Monoctonussimilis*
–	Antenna with 15 antennomeres; pterostigma length/width ratio about 5.7, pterostigma length/R1 vein ratio about 3.2, 2RS vein visible in half, m-cu vein distinct	*Monoctonuskoreanus* **sp. nov.**

*M.similis* is known only from type material.

## Analysis

**Figure 2.**
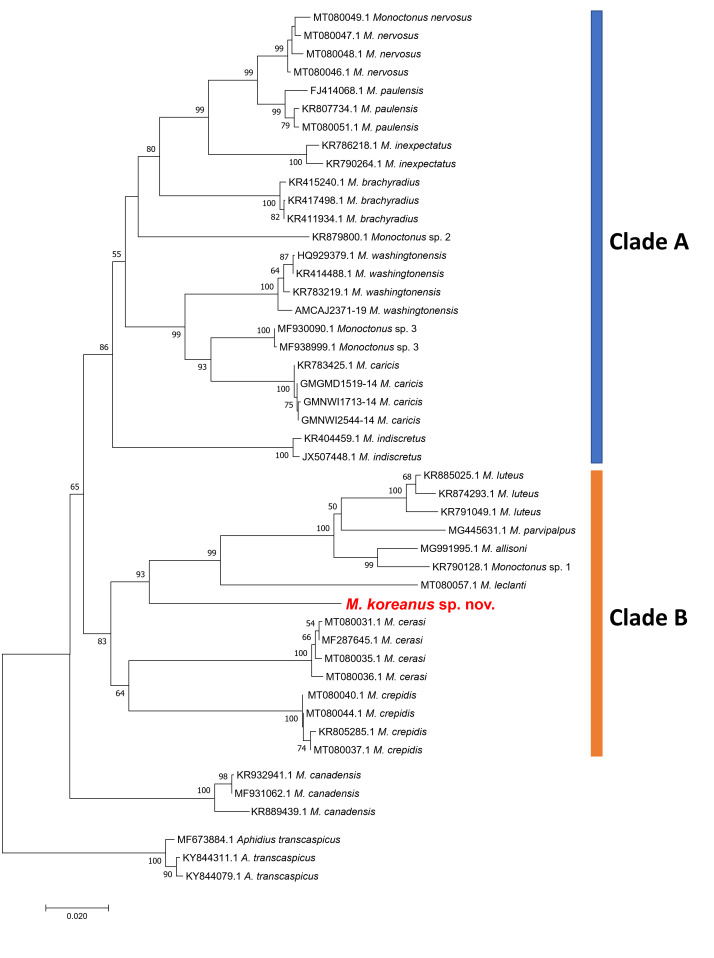
Neighbor-joining tree of 18 *Monoctonus* spp., based on their *COI* DNA barcodes. *Aphidiustranscaspicus* was used as outgroup. Bootstrap support values over 50% are indicated above branches. Scale bar means the expected rate of nucleotide substitution.

Amongst three ingroup clades, the NJ tree using *COI* barcoding data contains two main clades, A and B (Fig. 2). Clade A is subsequently subdivided into two subclades. *Monoctonusnervosus*, *M.paulensis*, *M.inexpectatus*, *M.brachyradius* and *Monoctonus* sp. 2 are clustered together, forming a sister clade to the group comprising *M.washingtonensis*, *Monoctonus* sp. 3 and *M.caricis*. *Monoctonusindiscretus* is also in clade A, forming a separate branch. Clade B is also subdivided into two subclades. *Monoctonusluteus*, *M.parvipalpus*, *M.allisoni*, *Monoctonus* sp. 1, *M.leclanti* and *M.koreanus* sp. nov., which is located in a basal position, are clustered together, joining a subclade consisting of *M.cerasi* and *M.crepidis*. *Monoctonuscanadensis* forms a separate clade sister to the clades A and B (Fig. 2). This result strongly supports the speciation of *M.koreanus* sp. nov. because it is independently located from the other species taxa in the tree.

Intraspecific and interspecific distances, based on the barcode region, range from 0 to 1.4% base pairs of difference (averaging 0.8%) and 2.7 to 19.5% (averaging 14.1%), respectively. Distances between the new species, *M.koreanus* sp. nov., and morphologically similar species, *M.nervosus*, *M.brachyradius* and *M.paulensis*, are 14.9%, 14.9% and 15.4%, respectively (Suppl. material 2).

## Supplementary Material

XML Treatment for Monoctonus koreanus

004DB5CB-1C54-5BAE-B449-EC143A38E090 10.3897/BDJ.12.e119476.suppl1 Supplementary material 1 Analysis sample list Data type Table File: oo_1006196.docx https://binary.pensoft.net/file/1006196 Sangjin Kim, Jelisaveta Čkrkić, Željko Tomanović, Ju-Hyeong Sohn, Jongok Lim and Hyojoong Kim

C7EA47DC-B754-5984-9055-454076BDA7A3 10.3897/BDJ.12.e119476.suppl2 Supplementary material 2 Calculated genetic distances Data type Table Brief description Calculated genetic distances, based on COI sequences between species of *Monoctonus* used in the analysis. File: oo_967521.docx https://binary.pensoft.net/file/967521 Sangjin Kim, Jelisaveta Čkrkić, Željko Tomanović, Ju-Hyeong Sohn, Jongok Lim and Hyojoong Kim
