# Supplementary material for: A new species of genus Monoctonus (Hymenoptera, Braconidae, Aphidiinae) from South Korea
Source: Biodivers Data J. 2024 Apr 15;12:e119476. doi: 10.3897/BDJ.12.e119476 (PMC11035975; doi:10.3897/BDJ.12.e119476)
Supplement: Supplementary material 1 — Analysis sample list [file bdj-12-e119476-s001.docx]

Table S1. Analysis sample list

| No | Species | NCBI accession number | BOLD ID |
| --- | --- | --- | --- |
| 1 | *Monoctonus koreanus* sp. n. | OK641850.1 |  |
| 2 | *M. allisoni* | MG991995.1 | GBAH23296-19 |
| 3 | *M. brachyradius* | KR417498.1 | CNMIJ605-14 |
| 4 | *M. brachyradius* | KR411934.1 | CNMIM048-14 |
| 5 | *M. brachyradius* | KR415240.1 | CNMIM742-14 |
| 6 | *M. canadensis* | KR932941.1 | CNGAB294-15 |
| 7 | *M. canadensis* | KR889439.1 | SSBAB2476-12 |
| 8 | *M. canadensis* | MF931062.1 | SSGIC2756-15 |
| 9 | *M. caricis* | KR783425.1 | CNGIJ830-13 |
| 10 | *M. caricis* | - | GMGMD1519-14 |
| 11 | *M. caricis* | - | GMNWI1713-14 |
| 12 | *M. caricis* | - | GMNWI2544-14 |
| 13 | *M. cerasi* | MT080035.1 | GBMNB62291-20 |
| 14 | *M. cerasi* | MT080031.1 | GBMNB62377-20 |
| 15 | *M. cerasi* | MT080036.1 | GBMNB62476-20 |
| 16 | *M. cerasi* | MF287645.1 | GBAH20742-19 |
| 17 | *M. crepidis* | KR805285.1 | CNGLC157-13 |
| 18 | *M. crepidis* | MT080037.1 | GBMNB62379-20 |
| 19 | *M. crepidis* | MT080040.1 | GBMNB62389-20 |
| 20 | *M. crepidis* | MT080044.1 | GBMNB62396-20 |
| 21 | *M. indiscretus* | KR404459.1 | CNPEA1225-14 |
| 22 | *M. indiscretus* | JX507448.1 | GBAHB332-13 |
| 23 | *M. inexpectatus* | KR786218.1 | CNRMD2282-12 |
| 24 | *M. inexpectatus* | KR790264.1 | MBIOD839-13 |
| 25 | *M. leclanti* | MT080057.1 | - |
| 26 | *M. luteus* | KR791049.1 | CNKTA917-14 |
| 27 | *M. luteus* | KR885025.1 | SSBAB2488-12 |
| 28 | *M. luteus* | KR874293.1 | SSBAB2502-12 |
| 29 | *M. nervosus* | MT080049.1 | GBMNB62382-20 |
| 30 | *M. nervosus* | MT080046.1 | GBMNB62397-20 |
| 31 | *M. nervosus* | MT0800471. | GBMNB62398-20 |
| 32 | *M. nervosus* | MT080048.1 | GBMNB62401-20 |
| 33 | *M. parvipalpus* | MG445631.1 | POSPD887-15 |
| 34 | *M. paulensis* | KR807734.1 | CNGII374-13 |
| 35 | *M. paulensis* | FJ414068.1 | DSWAS387-07 |
| 36 | *M. paulensis* | MT080051 | GBMNB60959-20 |
| 37 | *M. washingtonensis* | - | AMCAJ2371-19 |
| 38 | *M. washingtonensis* | HQ929379.1 | BBHYF017-10 |
| 39 | *M. washingtonensis* | KR783219.1 | CNGLF2022-13 |
| 40 | *M. washingtonensis* | KR414488.1 | CNKOU231-14 |
| 41 | *Monoctonus* sp. 1 | KR790128.1 | CNJAC589-12 |
| 42 | *Monoctonus* sp. 2 | KR879800.1 | SSBAB025-12 |
| 43 | *Monoctonus* sp. 3 | MF930090.1 | SSKUB5220-15 |
| 44 | *Monoctonus* sp. 3 | MF938999.1 | SSKUB1168-15 |
| 45 | *Aphidius transcaspicus* | MF673884.1 | - |
| 46 | *Aphidius transcaspicus* | KY844311.1 | - |
| 47 | *Aphidius transcaspicus* | KY844079.1 | - |
