# Supplementary material for: A new species of genus Monoctonus (Hymenoptera, Braconidae, Aphidiinae) from South Korea
Source: Biodivers Data J. 2024 Apr 15;12:e119476. doi: 10.3897/BDJ.12.e119476 (PMC11035975; doi:10.3897/BDJ.12.e119476)
Supplement: Supplementary material 2 — Calculated genetic distances [file bdj-12-e119476-s002.docx]

**Table S2.** Calculated genetic distances based on COI sequences between species of *Monoctonus* used in the analysis

|  | *M. koreanus* sp. n. | *M. allisoni* | *M. brachyradius*  (n=3) | *M. canadensis*  (n=3) | *M. caricis*  (n=4) | *M. cerasi*  (n=4) | *M. crepidis*  (n=4) | *M. indiscretus*  (n=2) | *M. inexpectatus*  (n=2) | *M. leclanti* | *M. luteus*  (n=3) | *M. mervosus*  (n=4) | *M. parvipalpus* | *M. paulensis*  (n=3) | *M. washingtonensis*  (n=4) | *Monoctonus* sp. 1 | *Monoctonus* sp. 2 | *Monoctonus* sp. 3  (n=2) |
| --- | --- | --- | --- | --- | --- | --- | --- | --- | --- | --- | --- | --- | --- | --- | --- | --- | --- | --- |
| *M. koreanus* sp. n. | (0.000) |  |  |  |  |  |  |  |  |  |  |  |  |  |  |  |  |  |
| *M. allisoni* | 0.143 | (0.000) |  |  |  |  |  |  |  |  |  |  |  |  |  |  |  |  |
| *M. brachyradius* | 0.149 | 0.175 | (0.002) |  |  |  |  |  |  |  |  |  |  |  |  |  |  |  |
| *M. canadensis* | 0.146 | 0.164 | 0.134 | (0.011) |  |  |  |  |  |  |  |  |  |  |  |  |  |  |
| *M. caricis* | 0.141 | 0.184 | 0.097 | 0.122 | (0.000) |  |  |  |  |  |  |  |  |  |  |  |  |  |
| *M. cerasi* | 0.142 | 0.158 | 0.131 | 0.132 | 0.136 | (0.004) |  |  |  |  |  |  |  |  |  |  |  |  |
| *M. crepidis* | 0.137 | 0.156 | 0.115 | 0.128 | 0.142 | 0.116 | (0.001) |  |  |  |  |  |  |  |  |  |  |  |
| *M. indiscretus* | 0.146 | 0.181 | 0.112 | 0.130 | 0.125 | 0.146 | 0.140 | (0.005) |  |  |  |  |  |  |  |  |  |  |
| *M. inexpectatus* | 0.154 | 0.188 | 0.087 | 0.140 | 0.116 | 0.142 | 0.132 | 0.125 | (0.009) |  |  |  |  |  |  |  |  |  |
| *M. leclanti* | 0.151 | 0.124 | 0.176 | 0.160 | 0.184 | 0.161 | 0.165 | 0.186 | 0.174 | (0.000) |  |  |  |  |  |  |  |  |
| *M. luteus* | 0.146 | 0.060 | 0.178 | 0.170 | 0.188 | 0.159 | 0.160 | 0.191 | 0.184 | 0.130 | (0.014) |  |  |  |  |  |  |  |
| *M. mervosus* | 0.149 | 0.173 | 0.090 | 0.119 | 0.120 | 0.137 | 0.137 | 0.119 | 0.067 | 0.173 | 0.175 | (0.007) |  |  |  |  |  |  |
| *M. parvipalpus* | 0.154 | 0.058 | 0.181 | 0.177 | 0.195 | 0.170 | 0.155 | 0.189 | 0.188 | 0.137 | 0.061 | 0.188 | (0.000) |  |  |  |  |  |
| *M. paulensis* | 0.154 | 0.174 | 0.088 | 0.123 | 0.111 | 0.137 | 0.133 | 0.126 | 0.063 | 0.173 | 0.176 | 0.027 | 0.185 | (0.009) |  |  |  |  |
| *M. washingtonensis* | 0.153 | 0.177 | 0.102 | 0.124 | 0.065 | 0.148 | 0.137 | 0.114 | 0.120 | 0.168 | 0.179 | 0.116 | 0.181 | 0.105 | (0.006) |  |  |  |
| *Monoctonus* sp. 1 | 0.141 | 0.029 | 0.170 | 0.176 | 0.177 | 0.163 | 0.165 | 0.186 | 0.182 | 0.126 | 0.065 | 0.180 | 0.063 | 0.180 | 0.175 | (0.000) |  |  |
| *Monoctonus* sp. 2 | 0.150 | 0.165 | 0.099 | 0.150 | 0.113 | 0.143 | 0.137 | 0.118 | 0.113 | 0.175 | 0.178 | 0.108 | 0.176 | 0.112 | 0.114 | 0.164 | (0.000) |  |
| *Monoctonus* sp. 3 | 0.148 | 0.181 | 0.090 | 0.130 | 0.047 | 0.137 | 0.140 | 0.120 | 0.101 | 0.184 | 0.180 | 0.107 | 0.183 | 0.095 | 0.067 | 0.177 | 0.105 | (0.000) |
